# Room temperature CO_2_ reduction to solid carbon species on liquid metals featuring atomically thin ceria interfaces

**DOI:** 10.1038/s41467-019-08824-8

**Published:** 2019-02-26

**Authors:** Dorna Esrafilzadeh, Ali Zavabeti, Rouhollah Jalili, Paul Atkin, Jaecheol Choi, Benjamin J. Carey, Robert Brkljača, Anthony P. O’Mullane, Michael D. Dickey, David L. Officer, Douglas R. MacFarlane, Torben Daeneke, Kourosh Kalantar-Zadeh

**Affiliations:** 1 School of Engineering, RMIT University, Melbourne, VIC 3001 Australia; 2 Graduate School of Biomedical Engineering, University of New South Wales (UNSW), Sydney, NSW 2052 Australia; 3 College of Material Science and Technology, Nanjing University of Aeronautics and Astronautics, 29 Jiangjun Ave, 211100 Nanjing, China; 4 School of Chemical Engineering, University of New South Wales (UNSW), Sydney, NSW 2031 Australia; 5 ARC Centre of Excellence for Electromaterials Science and Intelligent Polymer Research Institute, University of Wollongong, Wollongong, NSW 2522 Australia; 6 Institute of Physics and Center for Nanotechnology, University of Münster, Wilhelm-Klemm-Straße 10, 48149 Münster, Germany; 7 School of Science, RMIT University, Melbourne, VIC 3001 Australia; 8 School of Chemistry, Physics and Mechanical Engineering, Queensland University of Technology (QUT), Brisbane, QLD 4001 Australia; 9 Department of Chemical and Biomolecular Engineering, North Carolina State University, Raleigh, 27607 USA; 10 ARC Centre of Excellence for Electromaterials Science, Monash University, Clayton, VIC 3800 Australia

## Abstract

Negative carbon emission technologies are critical for ensuring a future stable climate. However, the gaseous state of CO_2_ does render the indefinite storage of this greenhouse gas challenging. Herein, we created a liquid metal electrocatalyst that contains metallic elemental cerium nanoparticles, which facilitates the electrochemical reduction of CO_2_ to layered solid carbonaceous species, at a low onset potential of −310 mV vs CO_2_/C. We exploited the formation of a cerium oxide catalyst at the liquid metal/electrolyte interface, which together with cerium nanoparticles, promoted the room temperature reduction of CO_2_. Due to the inhibition of van der Waals adhesion at the liquid interface, the electrode was remarkably resistant to deactivation via coking caused by solid carbonaceous species. The as-produced solid carbonaceous materials could be utilised for the fabrication of high-performance capacitor electrodes. Overall, this liquid metal enabled electrocatalytic process at room temperature may result in a viable negative emission technology.

## Abstract (web summary)

While CO_2_ reduction proves an appealing means to convert greenhouse emissions to high-value products, there are few materials capable of such a conversion. Here, the authors demonstrate a liquid-metal electrocatalyst to convert CO_2_ directly into solid carbon that can be used as capacitor electrodes.

## Introduction

Current projections for greenhouse gas emissions and global warming suggest that negative emission technologies capable of actively removing CO_2_ from the atmosphere will likely become a necessity to provide a stable climate for future generations^1,2^.

Capturing CO_2_ for underground storage has been discussed for decades, however, engineering challenges and concerns surrounding possible leaks have hampered implementation^3^. Chemical conversion of CO_2_ into non-volatile products, which could provide a permanent storage solution, can be considered as an alternative pathway^1^. Reducing CO_2_ to value-added chemicals that can be either used as fuels themselves or as feedstock for chemical industries using renewable energy sources, has been identified as an ideal outcome for a carbon neutral future. However, the extraordinary scale of carbon emissions, with an estimated 40 Gt CO_2_ (giga-tonnes CO_2_) being currently emitted annually and a total emitted anthropogenic CO_2_ mass in the order of 1000s Gt CO_2_, casts doubt on the possibility of finding suitable value-added products that can fully mitigate current and past greenhouse gas emissions^3^. Instead, negative emission technologies that convert emitted anthropogenic CO_2_ into solid products that are suitable for indefinite storage are expected to play a crucial role in stabilising the global climate, once the current transition of the world’s economy to carbon neutral energy sources has been completed.

To effectively facilitate storage, the reduced products should be solids to avoid accidental release into the environment. Accordingly, the ideal catalyst ought to enable the reduction of CO_2_ to elemental carbon materials at high thermodynamic process efficiency, thereby reversing the combustion of fossil fuels at minimal energy consumption.

CO_2_ is a remarkably stable molecule and thus the design of CO_2_ reduction electrocatalysts that work at low overpotential and at room temperature is challenging. Two dominant approaches have been pursued to date, where CO_2_ is either reduced in its gaseous form at high temperature^4,5^ or dissolved CO_2_ is electrocatalytically reduced within a liquid environment^6–8^. The gaseous route typically relies on oxide catalysts that can reduce CO_2_ to CO at high temperatures^4,5^. Alternatively, liquid electrolyte-based electrochemistry can be used to produce a range of small molecules including CO, C_2_H_4_, CH_4_, HCO_2_H and CH_3_OH^6,9–11^. While these molecules are of commercial significance for chemical industries, they are not suitable when the indefinite removal of CO_2_ from the atmosphere on the Gt scale is the main objective, since they are also volatile, potent pollutants.

The reduction of CO_2_ to solid products is challenging, since any product may cover the catalyst’s surface through van der Waals adhesion, blocking access to catalytically active sites and causing damage to the catalyst in a process known as coking^12^. The term of coking describes the formation of carbonaceous materials that adhere to the surface of the catalyst and diminish catalytic activity^13^. As such, the term in its common use in the field of catalysis applies when (a) carbonaceous materials are produced and (b) these materials adhere to the surface. Recently introduced liquid metal (LM)-based catalysts have been shown to be remarkably resistant towards deactivation via coking^12^. The liquid nature of the catalyst prohibits any produced carbonaceous materials from adhering onto the surface during the course of a reaction by eliminating the impact of adhesive van der Waals forces between the by-products and the surface of the LM. As a result, LM-based electrocatalysts are expected to be ideally suited for the continuous reduction of CO_2_ to carbonaceous and graphitic products, since surface adhesion of the products and subsequent deactivation are expected to be a major challenge.

Gallium-based alloys are ideal targets for the design of LM-based electrocatalysts, since they remain liquid at room temperature, are non-toxic and are capable of dissolving most other metallic elements at concentrations suitable for catalysis^14^. Moreover, the bulk of LMs is devoid of oxygen, allowing the stabilisation of pyrophoric elements, such as rare-earth metals^15^, which could previously not be considered for CO_2_ catalysis in their zero valent state. The metallic nature of these liquids also ensures excellent conductivity which is crucial when designing electrocatalytic processes^14^. Common low-melting point gallium alloys, such as the eutectic mixture of Ga, In and Sn, referred to herein as galinstan, have however been found to be rather inactive catalysts when investigated for processes, such as electrochemical hydrogen evolution and CO_2_ reduction, leading to these alloys being largely unexplored for such applications^14,16,17^. Interestingly, the surface of LMs can be effectively tuned by alloying with other metallic elements^14,15^. When complex alloys containing multiple metallic elements are designed, the interfacial oxide of the LM is dominated by the oxide that provides the greatest reduction in Gibbs free energy^15^. These oxides typically form atomically thin, two dimensional (2D) interfacial layers^15^.

In this work, we demonstrate that this phenomenon may be exploited to produce highly active electrocatalysts for CO_2_ reduction. Cerium-containing LM were utilised as an electrocatalytic system, successfully converting CO_2_ to carbonaceous and graphitic products at room temperature. Here the reduction of Ce^3+^ to metallic Ce^0^ at a LM electrode occurs at comparatively low potentials that are in close vicinity of the standard reduction potentials of the CO_2_/CO and CO_2_/C couples^18–20^. Gaining access to Ce^0^ may also enable catalysis pathways that were previously inaccessible, due to the pyrophoric nature of Ce. Interestingly, the incorporated Ce appears in the form of nanoparticles that are found to enhance the catalytic process. In combination, these properties render LM-containing cerium (LMCe) as an intriguing target for the design of efficient CO_2_ reduction electrocatalysts. Here, we demonstrate the highly efficient reduction of CO_2_ to solid carbon on this material’s interface.

## Results

### Electrochemical conversion of CO_2_ using LMs

Synthesis of different weight fractions of metallic cerium (0.5, 1.0 and 3.0 wt%) into liquid galinstan was performed using a mechanical alloying approach (see Methods). Cerium containing LM was created, since cerium oxides are known to reduce CO_2_ to CO via the Ce^3+^–Ce^4+^ cycle^4,5^. Cerium’s solubility in liquid gallium and its alloys is expected to be between 0.1 and 0.5 wt%, while Ce_2_O_3_ is expected to dominate the LM surface, as a 2D layer, under ambient atmospheric conditions due to the high reactivity of cerium when compared to the constituents of galinstan, and the known oxidation mechanism of metallic cerium that leads to the initial formation Ce_2_O_3_ at the metal–air interface^15,21,22^.

**Fig. 1:**
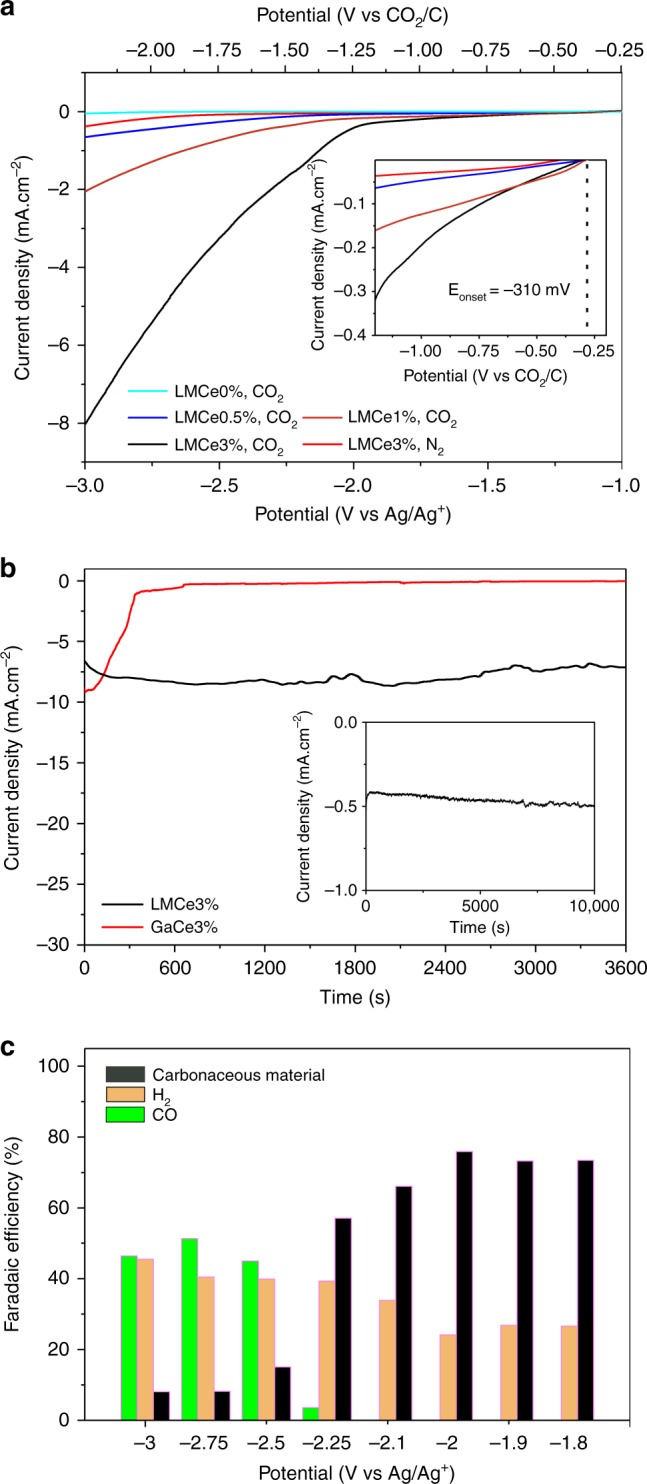
Characteristics of CO_2_ reduction by the LMCe electrocatalyst. **a** linear sweep voltammogram (LSV) of galinstan with different concentrations of Ce measured in 0.1 M tetrabutylammonium hexafluorophosphate (TBAPF_6_) and 2 M H_2_O in dimethylformamide (DMF) in N_2_ and CO_2_ saturated electrolyte. Inset shows a magnified view. **b** Chrono-amperometry results of liquid galinstan and solid gallium containing 3% Ce measured at −3 V vs. Ag/Ag^+^ in CO_2_ saturated electrolyte. Inset shows chrono-amperometry result of a liquid galinstan alloy containing 3 wt% Ce (LMCe3%) at −2 V vs. Ag/Ag^+^ in CO_2_ saturated electrolyte. Please note the Faradaic efficiency for the various products at −2 and −3 V vs. Ag/Ag^+^ which is shown in **c**. **c** Faradaic efficiencies of LMCe3% for the production of CO, H_2_, and solid carbonaceous material at corresponding potentials measured in CO_2_ saturated electrolytes. The Faradaic efficiency for the carbonaceous material was determined via a deduction process. Please refer to the Methods section for further details

The electrochemical reduction of CO_2_ using LMCe catalysts and pure LM (control) was conducted in a dimethylformamide (DMF)-based electrolyte, due to the high solubility of CO_2_ in the solvent^6^. Linear sweep voltammetry (LSV) was carried out utilising either CO_2_ or N_2_ (control) saturated electrolytes (Fig. 1a).

The cerium-containing alloys were able to support significant current densities and featured very low onset potentials (up to −310 mV vs. CO_2_/C) in the presence of CO_2_. The control experiment conducted in N_2_ atmosphere yielded negligible current densities (Fig. 1a). Consecutive cycles of saturating the electrolyte with N_2_ and CO_2_ were conducted (Supplementary Fig. 1) and a significant current density was only observed when the electrochemical tests were conducted in CO_2_ saturated electrolytes, demonstrating that the observed electrochemical processes are the result of the presence of the dissolved CO_2_. The experiments were found to be repeatable and close to identical current densities are observed in multiple subsequent cycles. The low current densities for N_2_ saturated electrolytes also indicate that the hydrogen evolution reaction, which is a competitive process to CO_2_ reduction, exhibits a relatively high overpotential on the LMCe electrode.

A further control experiment entailed conducting a typical CO_2_ reduction reaction in a different solvent, while also running a N_2_ saturated and CO_2_ free electrolyte-based control experiment (Supplementary Fig. 2). When acetonitrile was used, similar behaviour was observed as in the DMF-based experiment, indicating that the solvents are not likely taking part in the reaction.

In agreement with previous work, pristine galinstan was found to be a rather catalytically inactive electrode^16^. However, the activity of the alloy increased upon the addition of elemental cerium to the metallic melt. The observed current density correlates with increasing cerium content, and the onset potential for the most active LMCe3% electrode was found to be effectively −310 mV vs. CO_2_/C (Fig. 1a—inset and Supplementary Fig. 3). During the experiment, gas evolution was observed at higher applied potentials, indicating gaseous products. The inactivity of the cerium-free LM electrode in CO_2_ saturated electrolytes highlights the importance of cerium for the catalytic process.

### Characterisation of carbonaceous materials

**Fig. 2:**
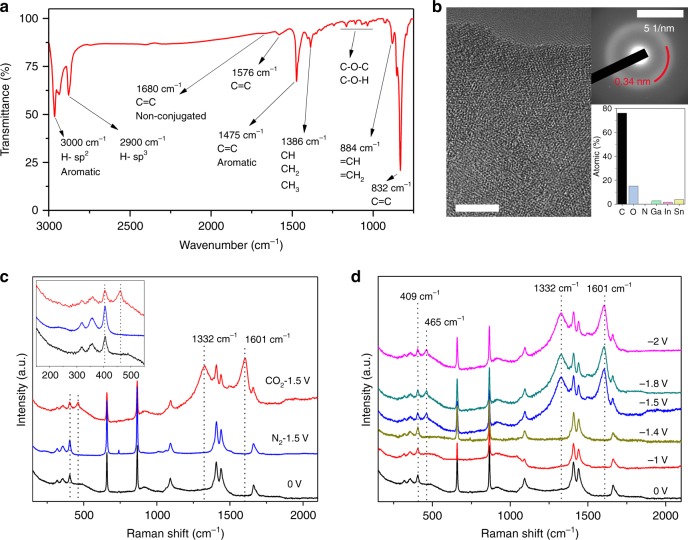
Characterisation of carbonaceous materials. **a** Fourier transform infra-red (FTIR) spectrum of the isolated carbonaceous materials, featuring intense FTIR absorption lines at 832 and 1475 cm^−1^ which are characteristic of C=C bonds. **b** High-resolution transmission electron microscopy (HRTEM) image of isolated layered carbonaceous materials (scale bar, 5 nm), with selected area electron diffraction (SAED) image (inset, scale bar 5 1/nm) and elemental composition determined by EDS (inset). **c** Raman spectroscopic measurement of carbonaceous materials on a liquid galinstan alloy containing 3 wt% Ce (LMCe3%) surface after electrochemical reduction in CO_2_ and N_2_ saturated electrolytes measured at 0 and −1.5 V vs. Ag/Ag^+^. Inset: magnified view of the Raman peaks at 409 and 465 cm^−1^. **d** *Operando* Raman spectra of the LMCe3% surface during electrocatalysis at indicated potentials

When CO_2_ was present in the electrolyte and a cerium-containing alloy was used, carbonaceous material could be produced which formed black floating debris in the electrolyte after prolonged electrolysis (Supplementary Fig. 4). The product was collected and purified for further analysis. Transmission electron microscopy (TEM, Fig. 2b and Supplementary Fig. 5) and scanning electron microscopy (SEM, Supplementary Fig. 6) analysis of these particulates revealed the appearance of small agglomerated flat sheets. High-resolution TEM (HRTEM) imaging and selected area electron diffraction (SAED) studies revealed an amorphous structure, indicating interatomic distances (0.34 nm) consistent with amorphous carbon (Fig. 2b)^23^. Atomic force microscopy (AFM, Supplementary Fig. 7) analysis of the produced carbonaceous nano-flakes found a typical thickness of 3 nm. Fourier transform infra-red (FTIR) spectroscopy (Fig. 2a and Supplementary Fig. 8) in combination with Raman spectroscopy (Fig. 2a) confirmed that the solid product is indeed predominantly composed of carbonaceous materials^19^. Similarly, the Raman spectrum reveals intense, broad features at 1332 and 1601 cm^−1^, which are characteristic of amorphous carbon sheets^23^. Furthermore, energy-dispersive X-ray (EDX) analysis revealed that the material is predominantly composed of carbon and oxygen, with insignificant quantities of the metal species present (Fig. 2b—bottom inset and Supplementary Fig. 9).

Elemental analysis of the products using X-ray photoelectron spectroscopy (XPS) was in agreement with the EDX results with the produced carbonaceous materials being mainly composed of carbon (84.49 at.%) while containing 14.99 at.% of oxygen (Supplementary Table 1). Small quantities of Sn are present and are likely associated with residual LM that was not successfully removed during the workup procedure. Detailed analysis of the C1*s* region of the XPS spectrum (Supplementary Fig. 10) revealed that the carbonaceous materials predominantly contains C–C and C=C bonds, while containing a significant fraction of covalently bound oxygen. FTIR analysis (Fig. 2a) also revealed the presence of C–H and C–O–H moieties. As such the obtained product is best described as amorphous carbonaceous nanosheets with a typical thickness of 3 nm.

Overall, the absence of a current response in the N_2_ control experiment, together with the isolated carbonaceous products indicate that the electrochemical process on the LMCe3% electrode was capable of converting gaseous CO_2_ into solid amorphous carbonaceous nanosheets at a low onset potential of only −310 mV vs. CO_2_/C, which is remarkable when considering the stability of the CO_2_ molecule. The control experiments (Supplementary Figs. 1–3), combined with a careful experimental design, allowed excluding the catalyst material, as well as the electrolyte as potential sources of the carbonaceous materials. The developed process occurred at room temperature, while previously developed electrocatalysts were only found to convert CO_2_ into solid products, such as carbon nanotubes, at very high temperatures (above 600 °C)^24,25^. A comparison of the onset potential and over potential for various CO_2_ reduction reactions in non-aqueous solutions (leading to gaseous and liquid products) is presented in Supplementary Table 2.

### Characterisation of the catalytic process

A detailed analysis of the electrochemical processes that occurred at the LMCe3% electrode was conducted and the Faradaic efficiencies for different products at various potentials were determined (Fig. 1c). Gas chromatography was employed to analyse the gaseous products. The Faradaic efficiency for the carbonaceous product was determined via a deduction process due to the challenges associated with the gravimetric analysis of small quantities of products that are generated during electrolysis (see discussion in the Methods section). As such, the determined efficiency is an upper estimate. However, electrochemical measurements in nitrogen saturated electrolytes (Fig. 1a) suggest that any parasitic processes (e.g. surface oxide reduction), and side reactions that may occur, are limited in magnitude and would have a small effect on the estimated Faradaic efficiency. The measurements revealed that solid carbonaceous materials were the dominant product at low potentials (faradaic efficiencies ~75% over the potential range −1.8 to −2.0 V vs. Ag/Ag^+^), while carbon monoxide becomes dominant at higher negative potentials.

The production of CO at more negative potentials likely occurs due to a separate process. The low potential region of the Tafel plot (Supplementary Fig. 11) reveals a distinct slow-moving process that occurs for reduction of CO_2_ to carbonaceous materials. Moderate quantities of hydrogen were produced as a side product. Nuclear magnetic resonance (NMR) spectroscopy was conducted on the electrolyte and revealed that small organic molecules were not produced (Supplementary Fig. 12). The presence of two parallel catalytic processes, which produce carbonaceous carbon in one instance and gaseous products in the second instance, renders the determination of an over-potential for the exclusive production of carbonaceous material producing reaction difficult. Therefore, the onset potential for the carbonaceous material producing process has been utilised herein.

The developed LMCe catalyst was observed to be stable during continued electrolysis experiments in either the higher potential region, where gas products are dominant (Fig. 1b), or the low potential region, where solid materials were produced (Fig.1b—inset). For comparison, an alloy containing 97% gallium and 3% cerium was synthesised, which remained solid at room temperature. Although, the solid electrode initially exhibited similar catalytic activity during CO_2_ electrolysis (Fig. 1b), the performance rapidly declined due to coking, highlighting that the liquid state of the electrode was crucial for continuous operation. The extraordinary stability of the liquid electrode may be associated with the lack of van der Waals adhesion on the liquid surface^12,14^. This observation leads to the conclusion that the processes, which result in carbonaceous products associated with deactivation via coking on solid catalysts can be exploited for continuously converting CO_2_ into solid products on LM electrodes.

*Operando* Raman spectroscopy was conducted to elucidate the operating mechanism of the catalyst. Figure 2d shows the Raman spectrum of the LMCe surface in the CO_2_ saturated electrolyte without any applied potential. Here the peak at 409 cm^−1^ is of particular significance since, it is characteristic for Ce_2_O_3_^26^, confirming that the surface of the LM contains significant amounts of Ce^3+^ ions. This is in excellent agreement with XPS measurements of the LMCe surface (Supplementary Fig. 13). The observation of Ce_2_O_3_ at the LM/air interface is consistent with oxidation studies on metallic cerium, which initially oxidised to form Ce_2_O_3_, which then partially converts to CeO_2_ after prolonged exposure to air (days)^27^.

Upon the application of reductive potential, additional peaks arise at 465, 1332 and 1601 cm^−1^ attributed to the formation of CeO_2_ and amorphous carbon species, respectively^23,28^. When a N_2_ saturated electrolyte was utilised, no new Raman peak emerged, confirming that the spectral changes were due to the CO_2_ reduction reaction (see also Supplementary Discussions for further details).

The presence of solid carbon species that arose due to an electrochemical reduction process and the emergence of CeO_2_, which resulted from the oxidation of Ce_2_O_3_ to CeO_2_, revealed critical insights into the catalytic mechanism.

The surface of the LMCe catalyst was initially dominated by Ce_2_O_3_ at room temperature. When a sufficiently negative electrochemical potential was applied, a portion of the surface Ce_2_O_3_ reduced to elemental Ce. Electrochemical studies on the LM electrode revealed that the onset of the Ce^3+^ reduction to Ce^0^ occurs at –1.2 V vs. Ag/Ag^+^ (Supplementary Fig. 14), which coincides with the onset potential of the electrocatalytic reaction at the LMCe catalyst. During electrocatalysis the zero-valent cerium atoms, which were produced, are capable of reacting with CO_2_ in a four-electron process, leading to the formation of CeO_2_ and carbonaceous products.

**Fig. 3:**
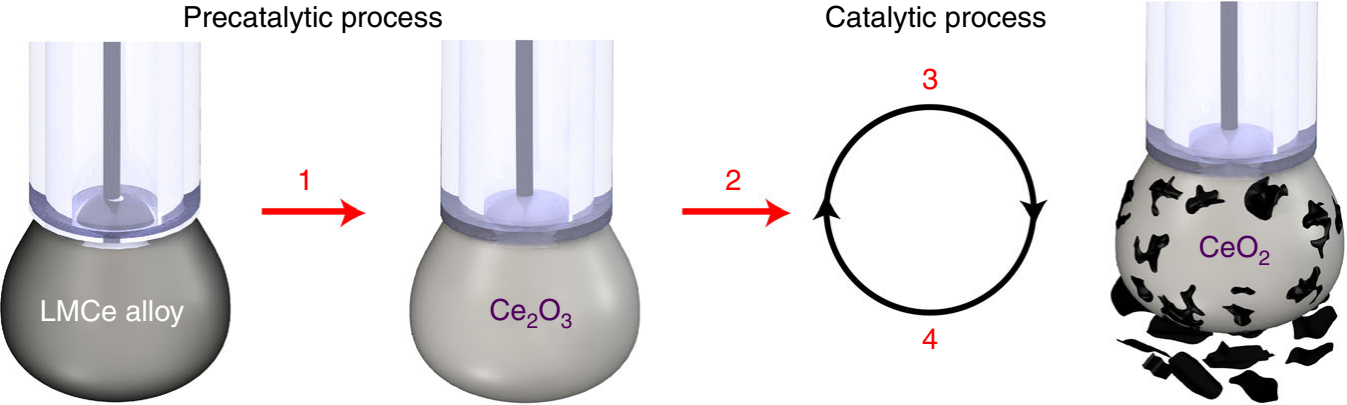
Schematic of the catalytic process. The proposed process is based on *operando* Raman measurements, it includes pre-catalytic reactions and the catalytic cycle for the CO_2_ reduction to amorphous carbon sheets. The picture is created by authors

Due to the applied reductive potential, the CeO_2_ was continuously reduced back to elemental Ce which drove the catalytic process. This correlates with the principle of the incipient hydrous oxide adatom mediator (IHOAM) model of electrocatalysis^29^. The process can be described by the chemical reactions 1–5. Reactions 1–4 are proposed to occur at the working electrode (Fig. 3), with reaction 5 describing the oxygen evolution reaction at the counter electrode.

1 $$2\,{\mathrm{Ce}}_{({\mathrm{Galinstan}})} + 1^{1/2}{\mathrm{O}}_{2({\mathrm{air}})} \to 2\,{\mathrm{Ce}}_2{\mathrm{O}}_3$$

2 $$2\,{\mathrm{Ce}}_2{\mathrm{O}}_3 + 3\,{\mathrm{H}}_2{\mathrm{O}} + 6\,{\mathrm{e}}^ - \to 2\,{\mathrm{Ce}}^{(0)} + 6\,{\mathrm{OH}}^ -$$

3 $${\mathrm{Ce}}^{(0)} + {\mathrm{CO}}_2 \to {\mathrm{CeO}}_2 + {\mathrm{C}}$$

4 $${\mathrm{CeO}}_2 + 2\,{\mathrm{H}}_2{\mathrm{O}} + 4\,{\mathrm{e}}^ - \to {\mathrm{Ce}} + 4\,{\mathrm{OH}}^ -$$

5 $$4\,{\mathrm{OH}}^ - \to {\mathrm{O}}_2 + 2\,{\mathrm{H}}_2{\mathrm{O}} + 4\,{\mathrm{e}}^ -$$

**Fig. 4:**
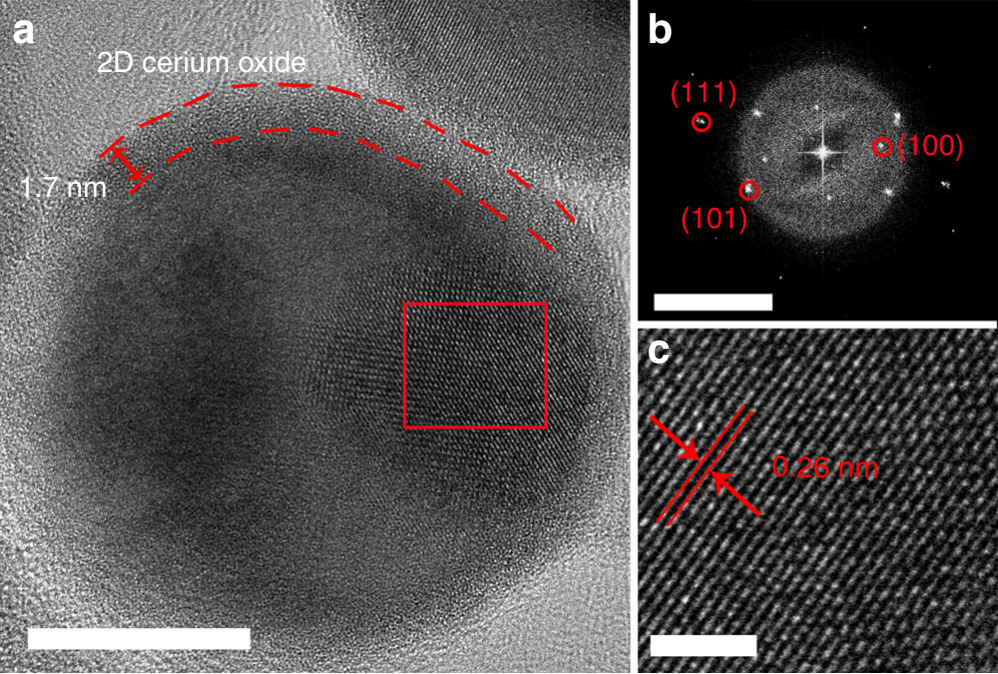
Characteristics of cerium oxide nanoparticles. **a** TEM image of a LMCe3% nanodroplet featuring encapsulated solid elemental cerium nanoparticles and an atomically thin layer of cerium oxide (scale bar, 10 nm). **b** FFT image of the crystalline section (scale bar 5 1/nm). **c** HRTEM image, the lattice parameters were indexed to elemental cerium (scale bar 2 nm)^30^

Cerium has a solubility limit between 0.1 and 0.5 wt% in liquid gallium^22^. Considering that 3 wt% Ce in LM leads to the best performing catalyst despite exceeding the solubility limit, further analysis was required. HRTEM analysis of LMCe droplets (Fig. 4) revealed the formation of a 2D cerium oxide layer with a thickness of ~1.7 nm on the LM surface. Furthermore, it is also seen that the excess Ce is present in the form of metallic nanoparticles, which are embedded within the LM. Analysis of the interatomic spacings in HRTEM images utilising fast Fourier transform (FFT) revealed that the crystalline solid inside the LM is elemental Ce^30^. The formation of Ce nanoparticles is notable due to the pyrophoric nature of the element. Their emergence is enabled because of the oxygen free environment within the LM. The presence of these solid inclusions facilitates the catalytic process by serving as a Ce source near the interface (Fig. 4).

### Electrode fabrication from carbonaceous materials

**Fig. 5:**
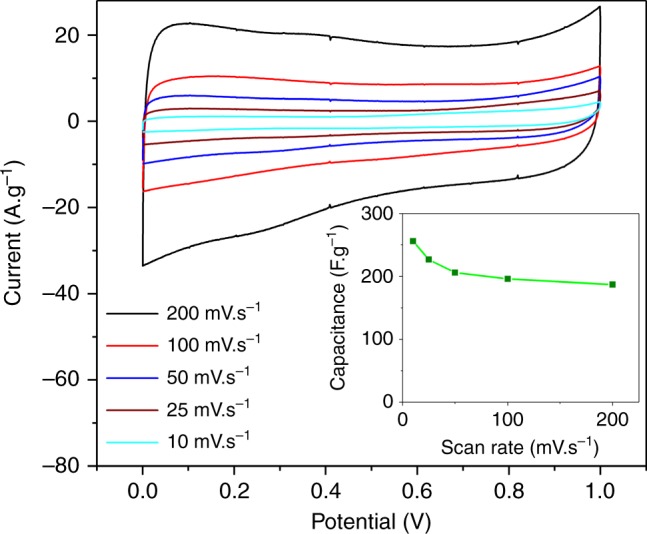
Supercapacitor behaviour of carbonaceous materials collected from CO_2_ conversion. Cyclic voltammograms of a double layer capacitor fabricated from synthesised carbonaceous materials in H_2_SO_4_ (1 M) electrolyte. Calculated specific capacitance of the capacitor at various scan rates (inset)

The isolated solid carbonaceous materials featured a highly porous superstructure as a result of an agglomerated plate-like morphology (Fig. 2b, Supplementary Figs. 5 and 6). Consequently, the collected carbonaceous product was fabricated into a two-electrode capacitor to show an example for the application of the by-products. The maximum capacitance of 250 F g^−1^ was recorded at 10 mV s^−1^, which is comparable to some of the best performing carbon-based supercapacitors in aqueous electrolytes^31^. These observations place the developed synthesis route among the most competitive techniques for producing high performance electrode materials using low-cost precursors under ambient condition (Fig. 5).

In the case of a future large-scale adoption of the developed process in the form of a negative emission technology, a portion of the produced carbonaceous materials may find application as electrode materials for energy storage applications; while any produced CO may be utilised as a feedstock for further industrial processes.

## Discussion

The process outlined here uses cerium containing LMs that feature 2D interfacial cerium oxide to enable the room temperature continuous electrocatalytic reduction of CO_2_ to solid carbon species at a very low onset potential (−1.2 V vs. Ag/Ag^+^; −310 mV vs. CO_2_/C). The solid carbonaceous materials are storable, enabling a negative CO_2_ emission technology when driven by renewable energy sources. Moreover, at higher applied potentials, CO was produced, which is a valuable precursor for a variety of industrial chemicals and synthetic fuels. The catalyst was remarkably resistant to deactivation via coking, due to the liquid state of the electrode, leading to limited van der Waals forces between the electrode and the carbonaceous product. The presence of metallic cerium nanoparticles within the alloy, above the solubility limit, was found to enhance the performance of the electrocatalysts significantly. Future works may utilise both electrode and electrolyte design to increase the activity of the catalyst further. Optimising the process will likely also improve the selectivity of the catalyst. Moreover, for the final application, nanodroplets and ultrathin LM films would be likely used to enhance surface area. Considering the short supply of some of the metals, such as In, the usage of higher melting Ga–Ce-based alloys, which only contain relatively abundant elements, may be considered for future works, provided that catalysis is conducted above the melting point of the alloy.

Overall, the findings are expected to lead to substantial future works, inspiring innovative LM-based catalyst designs for a variety of industrial processes, while also enabling selective and high-performance reaction designs that may take full advantage of the liquid state of metallic electrodes.

## Methods

### Galinstan and LMCe preparation

Eutectic Ga In Sn alloy (galinstan) was used as a base LM for producing LM mixtures that were used during this study. Gallium 99.99%, Indium 99.99% and Tin 99.9% were purchased from Roto Metals Inc. as precursors for galinstan. Galinstan was prepared in-house using previously established methods^15^, in order to avoid unknown additives which are commonly present in commercial galinstan. Galinstan was synthesised by melting 68.5 wt% gallium, 21.5 wt% indium and 10 wt% tin in a beaker on a hot plate at 250 °C. Upon complete melting, the liquid alloy was allowed to cool down to room temperature. Any surface oxides that have formed during the melting process was removed by transferring the produced LM from the beaker into a storage container using a pipette, by carefully taking the metal from the bulk liquid, leaving the oxidised surface behind.

In order to produce the cerium-containing catalytic mixtures, cerium powder (99.9% trace rare-earth metals basis, Sigma-Aldrich) was added to galinstan in concentrations of 0.5, 1 and 3 wt% inside a N_2_ glove box. The mixtures were ground using mortar and pestle until the alloy developed a smooth, reflective appearance, which indicated complete dissolution of the added cerium. This process typically took 15–30 min. Mechanical grinding using mortar and pestle led to the breakdown of the metal powders, removal of pre-existing surface oxides, while also increasing the interface between galinstan and cerium, effectively facilitating the dissolution of cerium.

Any surface oxides that may have formed during the grinding process, due to residual oxygen levels inside the glove box, were removed by transferring the produced LM from the mortar into a storage container using a pipette, as described above. The final LMCe alloys were kept in sealed vials inside the glove box until used. A control sample was prepared using pure gallium and 3 wt% Ce. The gallium was melted on a hot plate (50 °C) prior to the synthesis of the alloy.

### Electrode preparation and electrochemical measurements

The electrode was prepared by inserting conductive carbon fibres (World Precision Instruments) inside a glass capillary tube with 0.5 cm of the carbon fibre sticking out of the capillary. The fibre was then inserted into the LMCe alloy and through moving the capillary tube inside the liquid melt, a droplet of the LMCe could be attached to the carbon fibre at the end of the capillary tube. Care was taken to ensure that the end of the carbon fibre was physically isolated from the LM/electrolyte interface, eliminating the carbon fibre as a potential carbon source. The high surface tension of the LMCe allowed the droplet to maintain its shape during electrochemical measurements. The top end of the capillary tube was connected to electrochemical workstation using copper tape.

For further characterisations and measurements (i.e. continuous electrolysis), a drop of Ga–Ce alloy was collected using a plastic pipette and coated onto the entire surface of a glass sheet coated with conductive fluorine-doped tin oxide (FTO). The entire surface of FTO was covered with LM to avoid contact of FTO with electrolyte. Any measured values were normalised based on the geometric area of the LMCe droplets/LMCe-coated FTO surface area. For the measurement involving the solid Ga–Ce alloy, the metal was melted in a warm water bath (30 °C) and placed onto an entire sheet of FTO-coated glass. The sample was then allowed to cool down to room temperature and solidify. Full solidification typically occurred after 30 min of cooling. The FTO was visually inspected after the experiment, revealing that no changes occurred even after highly reducing potentials were applied. The minimal current density that was observed in the N_2_ control experiments highlight that any electrochemical process that may involve the FTO substrate is negligible.

Electrochemical measurements were performed in a low volume three-electrode cell (Pine Research Instrumentation, Inc.) including working, reference and counter electrode, using a potentiostat (CHI Instruments Inc., CHI760D) operated through CHI software version 14.04 USA.

The inlet of the electrochemical cells was connected to a CO_2_ gas cylinder, which contained CO_2_ with 99.98% purity (BOC). CO_2_ was purged with a high flow rate into the electrolyte for 30 min before any experiment was performed. During experiments CO_2_ was purged either at a slow rate into the electrolyte or into the headspace of the electrochemical cell (chronoamperometry measurements).

A BASI Pt counter electrode was utilised as a counter electrode and a silver/silver nitrate reference (10 mM AgNO_3_ in acetonitrile) served as a reference (BASI). The electrolyte contained 100 mM tetrabutylammonium hexafluorophosphate (TBAPF_6_, Sigma-Aldrich) and 2 M H_2_O in DMF (Sigma-Aldrich) and was prepared fresh before each measurement.

The electrolyte was collected for further analysis after the electrochemical reaction occurred. This could be achieved by removing the electrolyte close to the vicinity of the electrode, which was visually identified to contain the highest concentration of carbon particulates. Small droplets of LM were occasionally found to be transferred during this process. These LM inclusions could be effectively removed through repeated centrifugation (1 min at 2000 rcf). Centrifugation steps were repeated until no visible LM droplets could be identified. Carbon agglomerates remained suspended in the electrolyte and were analysed further during TEM-based and SEM-based characterisations.

### Instrumentation

LSV experiments were conducted at a sweep rate of 100 mV/s using the hanging drop electrodes. Chronoamperometry was performed at constant potentials of either −3 V vs. Ag/Ag^+^ or −2 V vs. Ag/Ag^+^ as indicated.

The electrochemically produced gases were analysed using gas chromatography (GC, Shimadzu, GC-08) with a thermal conductivity detector. The GC was equipped with a 6-foot molecular sieve 5 Å column using argon as carrier gas. The column was kept at 90 °C while the detector was heated to 100 °C for analysis. CO_2_ was continuously purged into an electrochemical cell and vented into the gas-sampling loop (5 mL) of the GC for gas analysis. The produced gases were calculated using calibration curves (CO: 6.30 × 1011 area mol^−1^, H_2_: 7.32 × 1012 area mol^−1^) determined by sampling known volumes of CO and H_2_ gas (see also Supplementary Fig. 15).

The electrochemical measurements during GC analysis were carried out using a potentiostat (CH Instruments, 650D, USA). The LMCe 3% alloy was drop-casted onto FTO glass, which served as the working electrode. A platinum mesh, isolated from the main chamber by a glass frit, was used as the counter electrode and an Ag/Ag^+^ (10 mM AgNO_3_ in acetonitrile) was used as the reference electrode. The measurements were conducted in an air-tight three electrode electrochemical cell for all GC experiments. Chronoamperometry was applied for each potential (−1.8 to −3.0 V vs. Ag/Ag^+^) for 20 min in the CO_2_-saturated electrolyte. For each potential, fresh working electrodes and electrolytes were used.

During the measurements, H_2_ and CO were the only detected produced gases, with no O_2_ evolution being observed (O_2_ was evolved at the physically isolated counter electrode). No liquid products were detected by NMR spectroscopy. The faradaic efficiency for H_2_ and CO production was calculated using Eq. (6), where *Q* is the charge passed during bulk electrolysis, *z* is the number of electrons consumed for producing a H_2_ or CO molecule, *n* is the number of moles of produced H_2_ or CO (determined using GC analysis), and *F* is the Faraday constant. The Faradaic efficiency for the carbonaceous material was determined via a deduction process, since the abundant presence of functional groups within the carbonaceous materials rendered the determination of *z* for this process difficult, while the produced mg quantities of carbonaceous products lead to error prone gravimetric quantification. The production of sufficient amounts of product to facilitate gravimetric analysis (i.e. 0.1–1 g) was determined to be impractical, since many days of electrolysis would be required to produce 1 g of carbonaceous product at current density of 1 mA/cm^2^ when using a 1 cm^2^ electrode (even considering an ideal case of a four electron reduction to elemental carbon and a 100% Faradaic efficiency). As such the above detailed approach was determined to be the most appropriate.

6 $${\mathrm{Faradaic}}\,{\mathrm{efficiency}}\left( {\mathrm{\% }} \right) = \frac{{{Q}_{{\mathrm{Experimental}}}}}{{{Q}_{{\mathrm{Theoretical}}}}} \times 100 = \frac{{{z} \times {n} \times {F}}}{{Q}} \times 100$$

### Material and morphological characterisations

In order to characterise the produced solid carbon species, the electrolyte was processed via centrifugation to remove any residual LM droplets. The solution was the dropped onto a TEM grid (lacey carbon grids, Prositech) and the solvent was allowed to evaporate. TEM imaging was conducted using a JEOL 2100F TEM/STEM (2011) instrument equipped with a Gatan OneView 4k CCD Camera. Measurements were conducted with a 200 keV acceleration voltage. TEM samples of LMs were prepared by sonicating the LMCe 3% alloy in DMF for 1 min at a sonication power of 100 W. The supernatant (after sonication) was directly dropped onto the TEM grid and used for HRTEM characterisation.

A FEI Verios 460L FEGSEM scanning electron microscope equipped with an Oxford X-Max20 EDX detector was used for the EDX analysis of the isolated carbonaceous materials.

Raman spectroscopy was conducted using a Horiba Scientific LabRAM HR evolution Raman spectrometer equipped with a ×100 objective lens, a 532 nm laser source (intensity 0.45 mW at the sample position) and a 1800 lines per mm grating. Measurements presented in Fig. 2c were achieved by running electrocatalysis for 20 min at defined potentials (either −1.5 V vs. Ag/Ag^+^ or at open circuit (0 V)) in defined environments (either N_2_ or CO_2_ saturated electrolytes). Raman spectroscopy was then conducted on the LM surface (no potential applied).

The presence of functional groups in the carbonaceous materials was investigated using microscope-based FTIR spectroscopy. Measurements were performed using a PerkinElmer Spectrum 100 FTIR Spectrometer connected to the Spotlight 400 FTIR Imaging System with Stage Controller. The carbonaceous products were identified and measured on the LM and a control spectrum measured on pristine LM was subtracted from the data.

XPS was conducted using a Thermo Scientific K-Alpha XPS spectrometer featuring a monochromated Al Kα X-ray source with photon energy of 1486.7 eV and an X-ray spot size of 30−400 μm. The sample was a small quantity of the LMCe 3% alloy smeared onto a Si substrate.

Solution state NMR spectra of the electrolyte were collected after prolonged CO_2_ reduction (4 h at −3 V vs. Ag/Ag^+^). Spectra were acquired on an Agilent DD2 500 MHz NMR spectrometer equipped with a 5 mm probe. 1 H NMR spectra were acquired with 1024 scans. The samples were analysed neatly, and a capillary containing D_2_O was placed into the NMR tube to allow for locking. Samples were referenced using the residual HDO peak (δH 4.64).

The active layer of the working electrodes was fabricated through deposition of 5 µg of the isolated carbonaceous product on glassy carbon electrodes (BASi) via drop casting. The carbonaceous materials were produced at −3 V vs. Ag/Ag^+^, allowing to produce sufficient product after extended electrolysis followed by sample purification (see above). The double layer capacitance was investigated using cyclic voltammetry (CV) via a potentiostat (CHI Instruments Inc., CHI760D) in a two-electrodes setup using H_2_SO_4_ (1 M) as an electrolyte.

### *Operando* Raman spectroscopy

In order to monitor the chemical changes during electrochemical reduction of CO_2_, Raman spectroscopy was performed under operation. A custom made electrochemical cells (ABS) was produced in-house using a laser milling machine to operate with a ×50 objective. The custom made electrochemical cell featured designated places for counter, reference and working electrode. The working electrode for this experiment was prepared on conductive carbon paper which was cut to size. The cell featured an electrolyte reservoir which was covered with a fitted 20 × 20 mm confocal fluorescence microscopy coverslip.

The electrolyte was purged with CO_2_ (control) for 30 min before any electrochemical measurement occurred, and the minimum volume of electrolyte was utilised in order to cover all three electrodes. The coverslip was inserted in a way to expulse any air bubbles. Raman measurements were conducted in-operando under applied potentials and the results are shown in Fig. 2d.

## Supplementary information

Supplementary Information

## Data Availability

All relevant data are available from the authors.
