# Publisher Correction: Room temperature CO_2_ reduction to solid carbon species on liquid metals featuring atomically thin ceria interfaces

**DOI:** 10.1038/s41467-019-09228-4

**Published:** 2019-03-20

**Authors:** Dorna Esrafilzadeh, Ali Zavabeti, Rouhollah Jalili, Paul Atkin, Jaecheol Choi, Benjamin J. Carey, Robert Brkljača, Anthony P. O’Mullane, Michael D. Dickey, David L. Officer, Douglas R. MacFarlane, Torben Daeneke, Kourosh Kalantar-Zadeh

**Affiliations:** 1 School of Engineering, RMIT University, Melbourne, VIC 3001 Australia; 2 Graduate School of Biomedical Engineering, University of New South Wales (UNSW), Sydney, NSW 2052 Australia; 3 College of Material Science and Technology, Nanjing University of Aeronautics and Astronautics, 29 Jiangjun Ave, 211100 Nanjing, China; 4 School of Chemical Engineering, University of New South Wales (UNSW), Sydney, NSW 2031 Australia; 5 ARC Centre of Excellence for Electromaterials Science and Intelligent Polymer Research Institute, University of Wollongong, Wollongong, NSW 2522 Australia; 6 Institute of Physics and Center for Nanotechnology, University of Münster, Wilhelm-Klemm-Straße 10, 48149 Münster, Germany; 7 School of Science, RMIT University, Melbourne, VIC 3001 Australia; 8 School of Chemistry, Physics and Mechanical Engineering, Queensland University of Technology (QUT), Brisbane, QLD 4001 Australia; 9 Department of Chemical and Biomolecular Engineering, North Carolina State University, Raleigh, 27607 USA; 10 ARC Centre of Excellence for Electromaterials Science, Monash University, Clayton, VIC 3800 Australia

Correction to: *Nature Communications*; 10.1038/s41467-019-08824-8; published online 26 February 2019

The original version of this Article contained errors in the author affiliations.

Affiliation 1 incorrectly read ‘School of Chemical Engineering, University of New South Wales (UNSW), Sydney, NSW 2031, Australia’ and affiliation 4 incorrectly read ‘School of Engineering, RMIT University, Melbourne, VIC 3001, Australia.’

This has now been corrected in both the PDF and HTML versions of the Article.

