# Supplementary material for: Room temperature CO2 reduction to solid carbon species on liquid metals featuring atomically thin ceria interfaces
Source: Nat Commun. 2019 Feb 26;10:865. doi: 10.1038/s41467-019-08824-8 (PMC6391491; doi:10.1038/s41467-019-08824-8)
Supplement: Supplementary file 1 — Supplementary Information [file 41467_2019_8824_MOESM1_ESM.pdf]

Supplementary Materials:

Room temperature CO_2 reduction to solid carbon species on liquid metals featuring atomically thin ceria interfaces

Dorna Esrafilzadeh[1,9*], Ali Zavabeti[1,10], Rouhollah Jalili[8], Paul Atkin[1], Jaecheol Choi[3], Benjamin J. Carey[4], Robert Brkljača[2], Anthony P. O'Mullane[5], Michael D. Dickey[6], David L. Officer[3], Douglas R. MacFarlane[7], Torben Daeneke[1*], Kourosh Kalantar-Zadeh[8,1*]

[1] School of Engineering, RMIT University, Melbourne, VIC, 3001, Australia

[2] School of Science, RMIT University, Melbourne, VIC, 3001, Australia

[3] ARC Centre of Excellence for Electromaterials Science and Intelligent Polymer Research Institute for Innovative Materials, University of Wollongong, Wollongong, NSW, 2522, Australia

[4] Institute of Physics and Center for Nanotechnology, University of Münster, Wilhelm-Klemm-Straße 10, 48149 Münster, Germany

[5] School of Chemistry, Physics and Mechanical Engineering, Queensland University of Technology (QUT), Brisbane, QLD, 4001, Australia

[6] Department of Chemical and Biomolecular Engineering, North Carolina State University, Raleigh, 27607, USA

[7] ARC Centre of Excellence for Electromaterials Science, Monash University, Clayton, VIC, 3800, Australia

[8] School of Chemical Engineering, University of New South Wales, Sydney, UNSW, 2031, Australia

[9] Graduate School of Biomedical Engineering, University of New South Wales (UNSW), Kensington, Sydney, NSW 2052, Australia

[10] College of Materials Science and Technology, Nanjing University of Aeronautics and Astronautics, 29 Jiangjun Ave., Nanjing, 211100, Nanjing, China

*Correspondence to: d.esrafilzadeh@unsw.edu.au; torben.daeneke@rmit.edu.au; k.kalantar-zadeh@unsw.edu.au

Supplementary Figures and Tables:

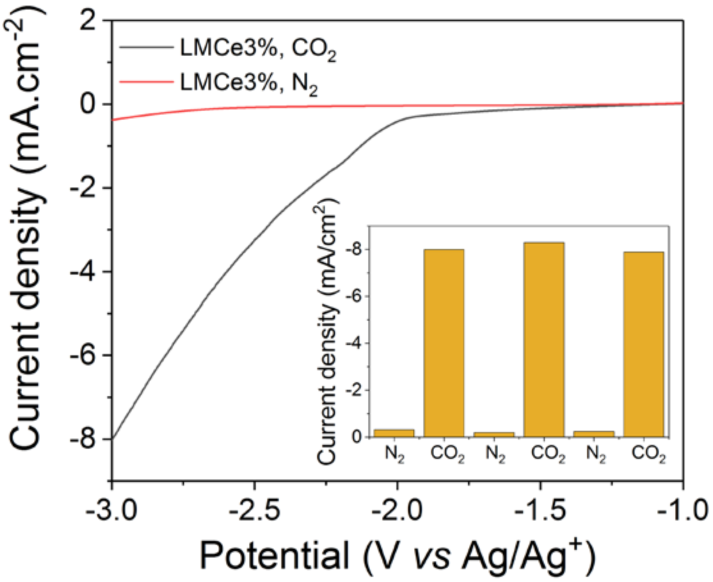

Supplementary Figure 1. LSV of galinstan with 3 wt% Ce measured in 0.1 M $TBAPF_6$ and 2M H_2O in DMF under N_2 and CO_2 saturated electrolyte. Inset shows the current density at -3V over 3 consecutive switching cycles between N_2 and CO_2.

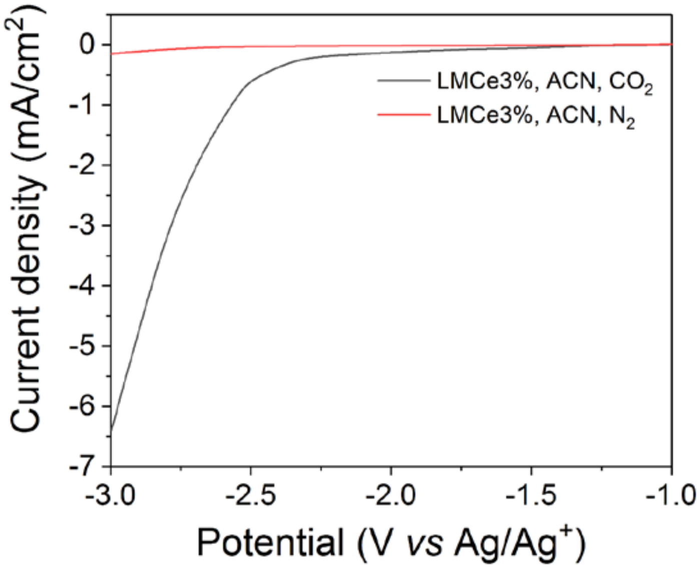

Supplementary Figure 2. LSV of galinstan with 3 wt% Ce measured in 0.1 M $TBAPF_6$ and 2M H_2O in ACN under N_2 and CO_2 saturated electrolyte.

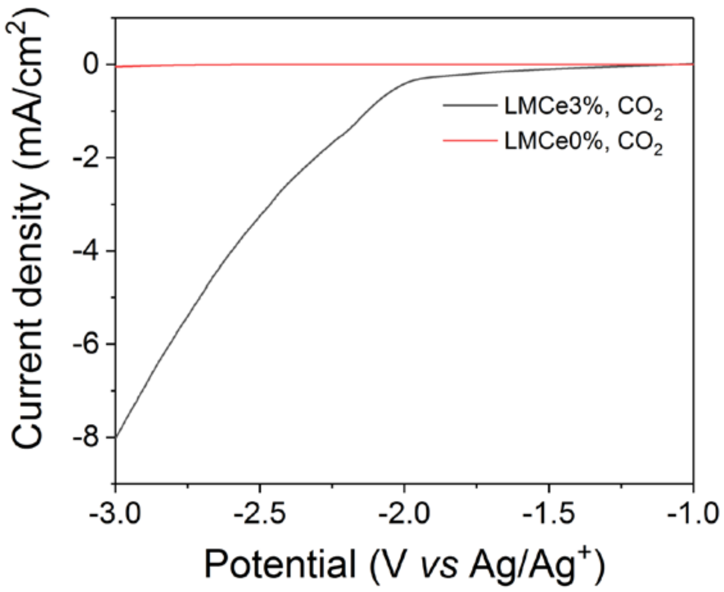

Supplementary Figure 3. LSV of galinstan with 0 wt% and 3 wt% Ce measured in 0.1 M $TBAPF_6$ and 2M H_2O in DMF under CO_2 saturated electrolyte.

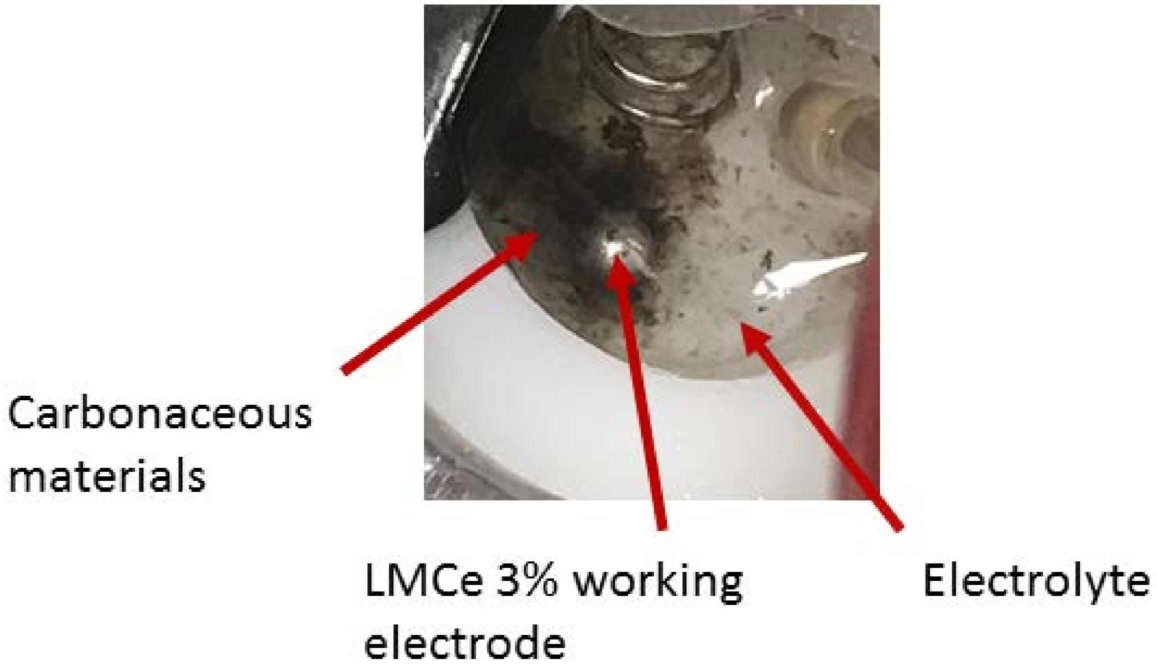

Supplementary Figure 4. Photograph of the electrochemical cell with liquid galinstan working electrode containing 3% cerium (LMCe3%) that shows delaminated carbonaceous materials.

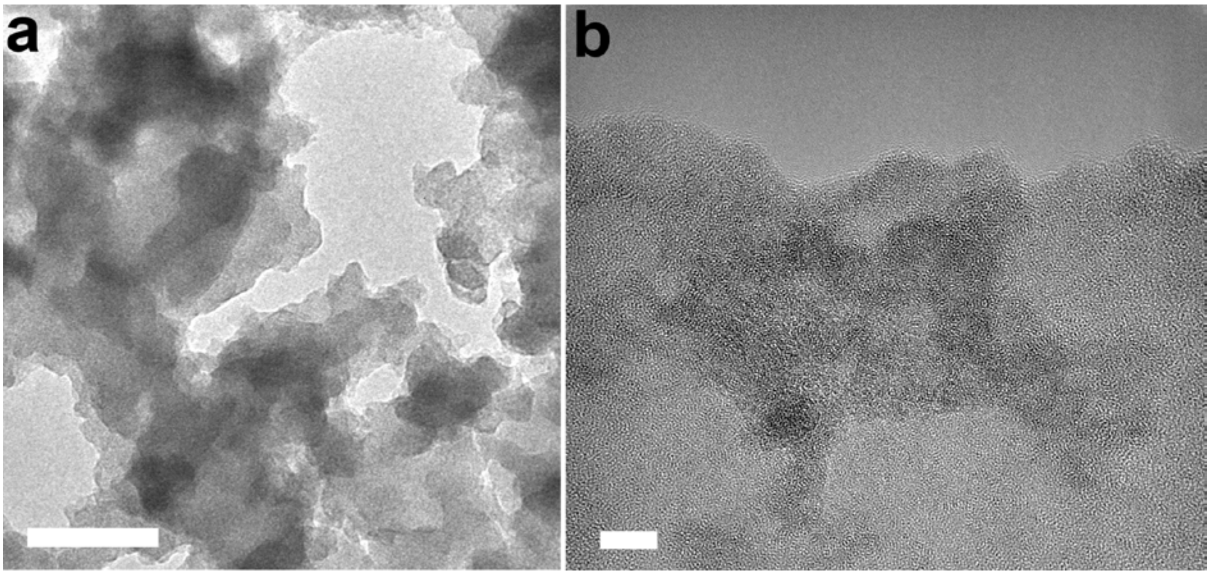

Supplementary Figure 5. TEM images of the produced carbonaceous material. **a** lower magnification (scale bar shows 50 nm). **b** Higher magnification (scale bar shows 5 nm).

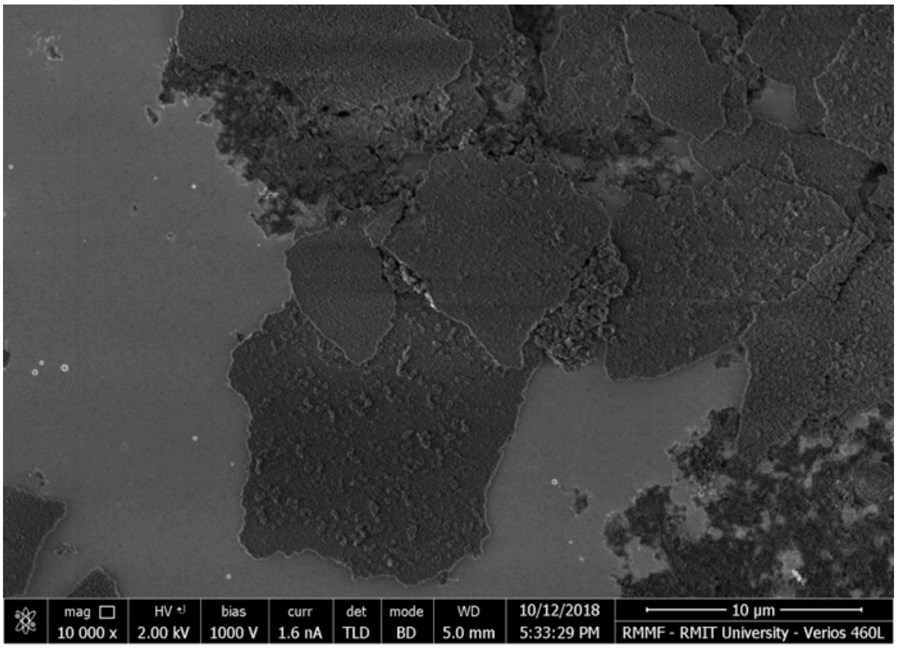

Supplementary Figure 6. SEM images of the produced carbonaceous materials.

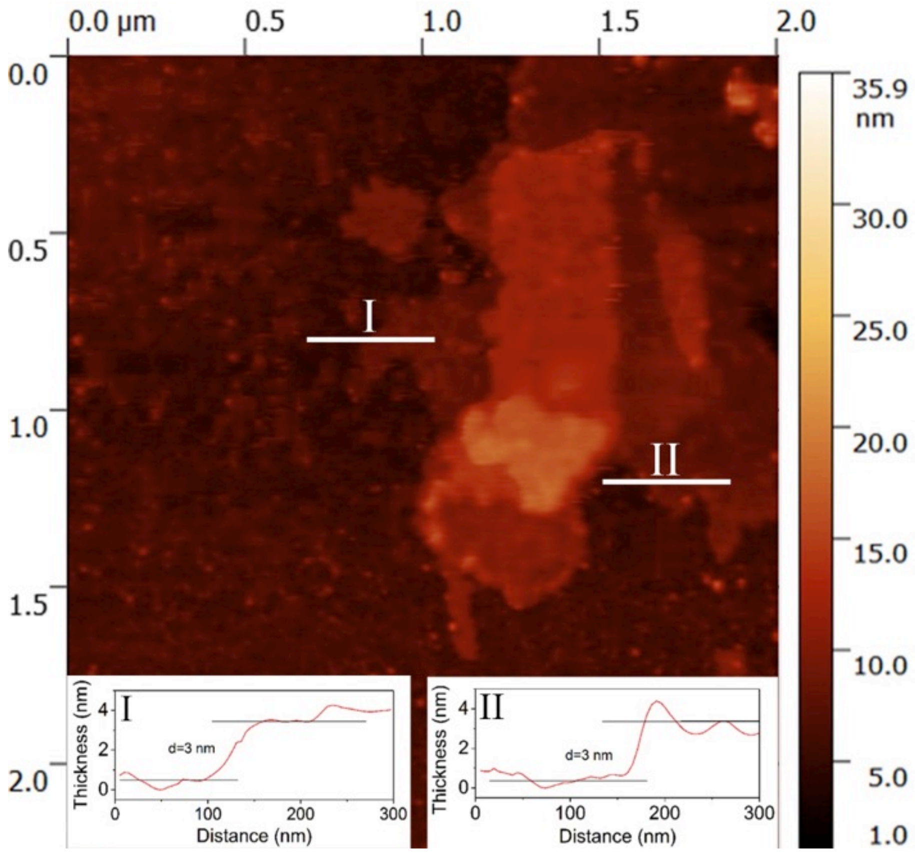

Supplementary Figure 7. Atomic Force Microscopy (AFM) analysis of carbonaceous materials showing the thickness of products was 3 nm.

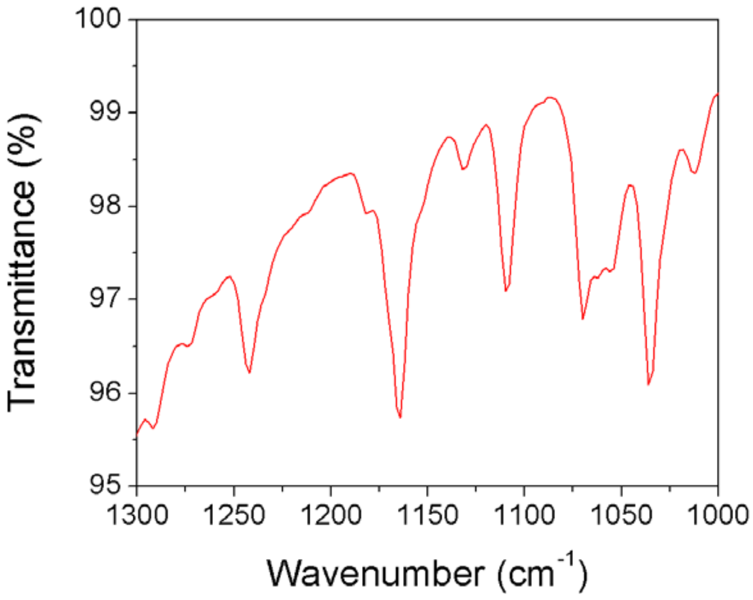

Supplementary Figure 8. FTIR spectrum of carbonaceous materials between 1000 to 1300 cm^{-1}. Smaller peaks at 884 and 1386 cm^{-1} indicate the presence of C-H bonds, and numerous small peaks between 1020 and 1250 cm^{-1} attest to the presence of some C-O-C and C-O-H moieties.

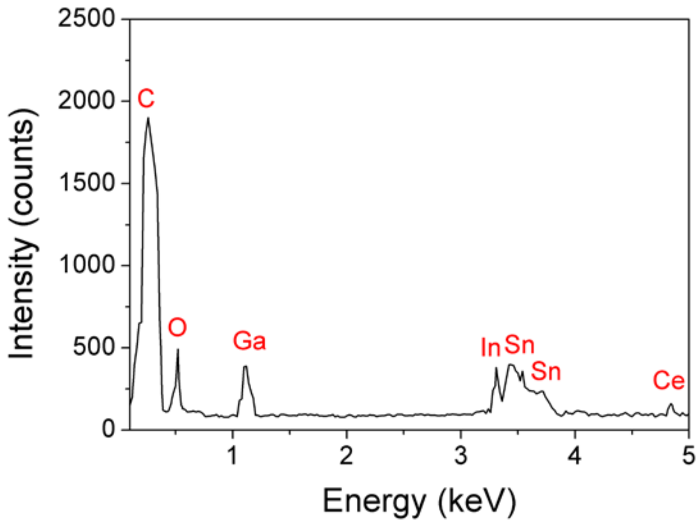

Supplementary Figure 9. EDS of carbonaceous materials. EDX analysis of the solid product did not show the presence of nitrogen and hence the nitrogen-containing solvent is unlikely to be a participant in the reaction.

Supplementary Table 1. Atomic percentage of each component calculated from XPS analysis of the isolated product.

Element	Atomic %
C	84.49
O	14.99
Ce	0.00
Ga	0.00
In	0.00
Sn	0.49

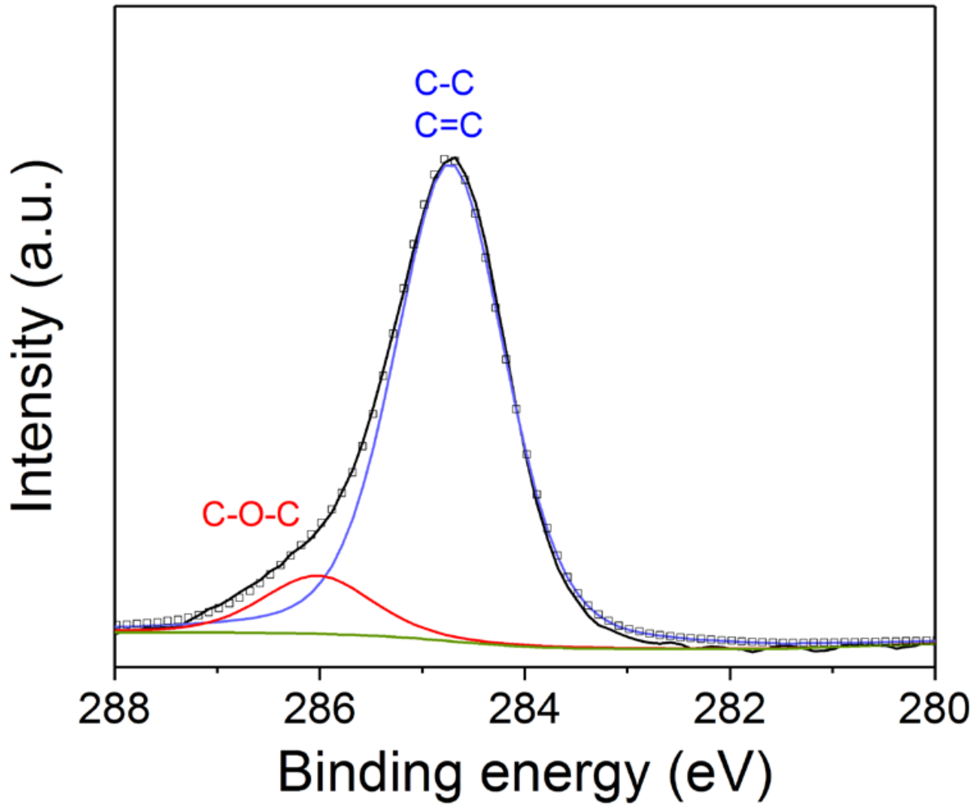

Supplementary Figure 10. X-ray photoelectron spectroscopy (XPS) of carbonaceous materials, which shows the C1s spectra of the carbonaceous materials, which is fitted to two peaks centred at 284.76 and 286.08 eV.

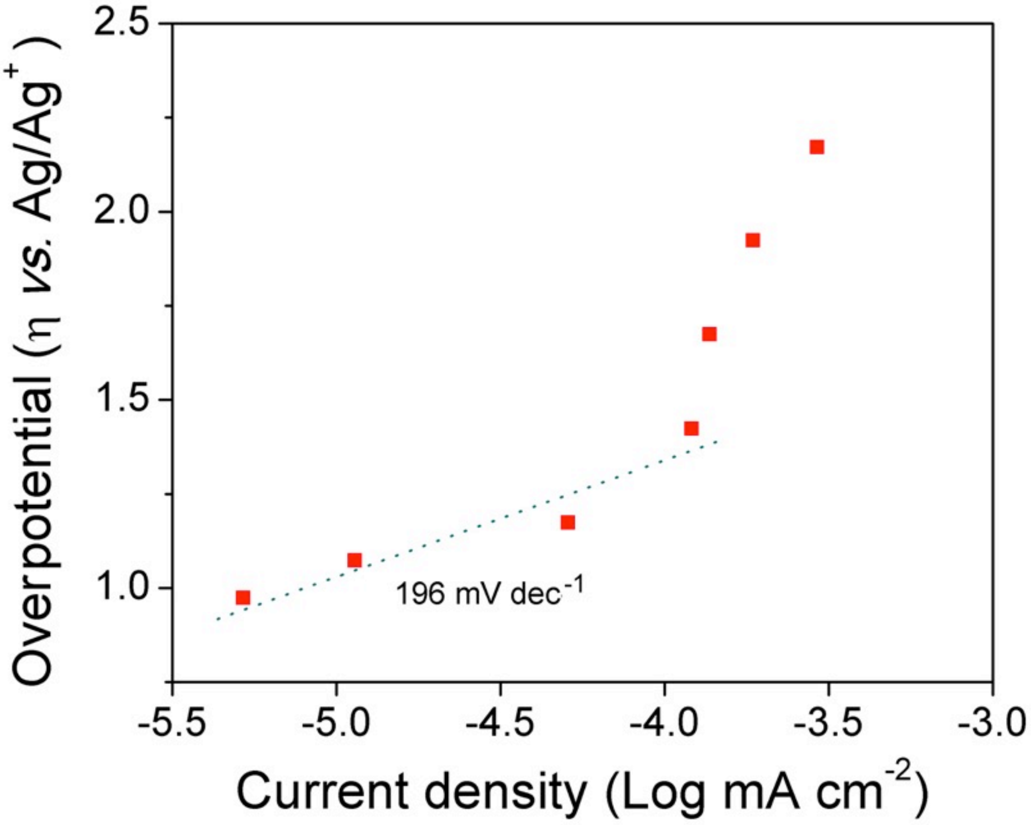

Supplementary Figure 11. Tafel plot of LMCe 3% under CO_2 environment.

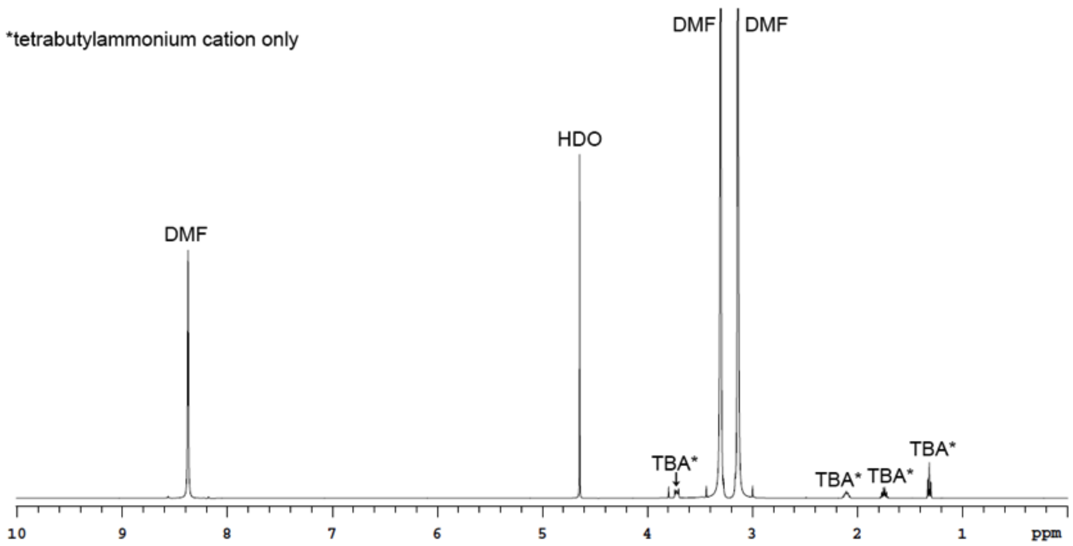

Supplementary Figure 12. NMR spectroscopy of the electrolyte after 4 hr of electrocatalysis.

Supplementary Table 2. Comparison of the over potential and onset potential for various CO_2 reduction reactions in non-aqueous solutions.

**Catalysts**	**Products**	**Electrolyte**	**Over potential for each product[1,3] (at 1mA/cm^2)**	**Onset potential for each product[1,3]**	**Ref.**
**LMCe 3%**	**Carbonaceous materials**	**DMF/2M H_2O (0.1M $TBAPF_6$)**	**-1.375 vs (CO_2/C)**	**-0.310 vs (CO_2/C)**	**This work**
**LMCe 3%**	**CO**	**DMF/2M H_2O (0.1M $TBAPF_6$)**	**NA**	**-1.016 vs (CO_2/CO)[2]**	**This work**
Zn(II) tetramesitylporphyrin	CO	DMF/10% H_2O (0.1M $TBAPF_6$)	-1.4 vs SHE	NA	1
Fe_2DTPFPP-PO_3H_2	CO	DMF/5% H_2O (0.1M $TBAPF_6$)	NA	-1.4 vs NHE (@ 0.7 mA/cm^2)	2
Re-grafted metal organic framework (MOF)	CO	MeCN/5% EtOH (0.1M TBAH)	-1.45 vs NHE	NA	3
Co_3O_4 nanofibres	CO	MeCN/1% H_2O (0.1M $TBAPF_6$)	NA	-1.76 vs NHE (@ 0.175 mA/cm^2)	4
Surface treated Bi nanoparticles (Bi NPs)	CO	MeCN/Ionic liquid	-1.7 vs Ag/AgCl	NA	5
Cu_2Pd alloy	CH_4	MeCN/1M H_2O (0.1M $TBAPF_6$)	NA	Onset -1.6 vs Ag/AgCl	6
Sn	HCOOH	MeCN/H_2O/Ionic liquid	-1.8 vs Ag/AgCl	NA	7
Pb	HCOOH	MeCN/H_2O/Ionic liquid	-2.0 vs Ag/AgCl	NA	7
Cu/Cu_2O	HCOOH	DMF/1% H_2O (0.1M $TBAPF_6$)	-2 vs Fc^+/Fc (@ 1 mA)	NA	8
SnO_2@N-doped porous carbon	HCOOH	MeCN/Ionic liquid	-2.0 vs Ag/AgCl	NA	9
Electrodeposited-[Cu(cyclam)]$(ClO_4)_2$	HCOOH	DMF/3% H_2O (0.1M $TBABF_4$)	0.15 vs Fc^+/Fc	NA	10

[1] The standard potentials of CO_2 reduction reactions vary considerable in different solvents and in the presence of proton donors. For many electrolyte systems these potentials are not known and readers should refer to the original works for further detail.

[2] Onset potential was determined by estimating the potential initially generating CO_2 reduction products. In the case of CO generated in this study, the first potential that could be directly measured using GC experiment is presented in the table as the onset potential.

[3] Equilibrium potentials: CO_2/CO=-0.69 V vs. NHE[11], CO_2/CH_4=-0.39 V vs. NHE[12] and CO_2/HCOOH=-0.78 V vs. NHE[13].

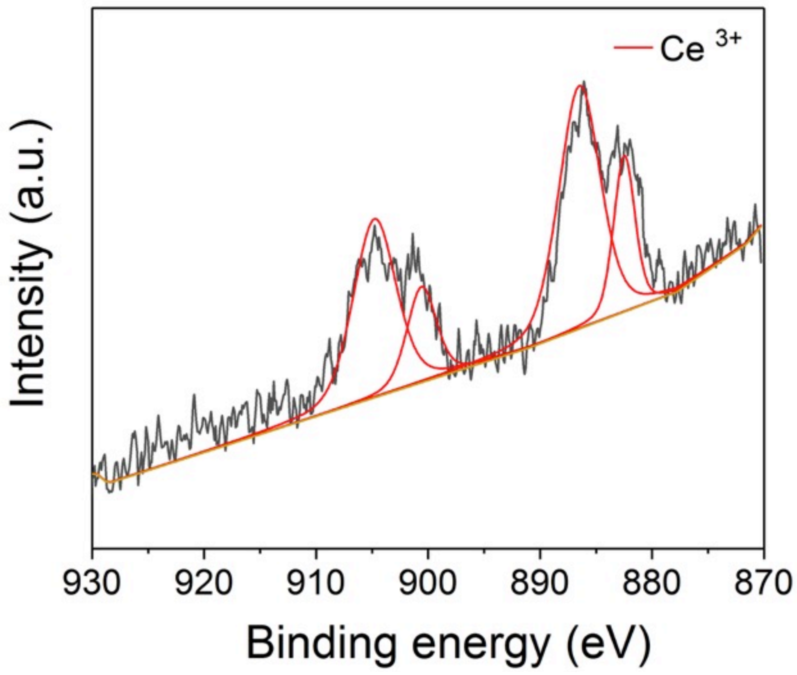

Supplementary Figure 13. XPS of the LMCe3% surface showing Ce^{3+} oxide peaks originating from the interface.

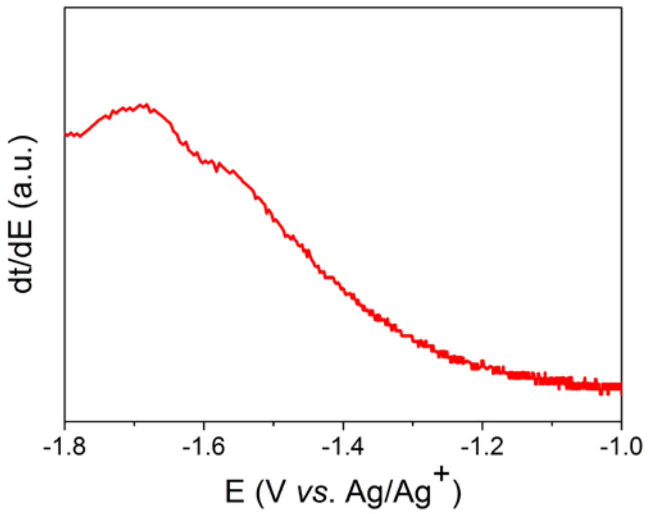

Supplementary Figure 14. Potentiometric stripping analysis of 1 mM cerium (III) nitrate hexahydrate in the electrolyte used during electrocatalysis on the galinstan electrode.

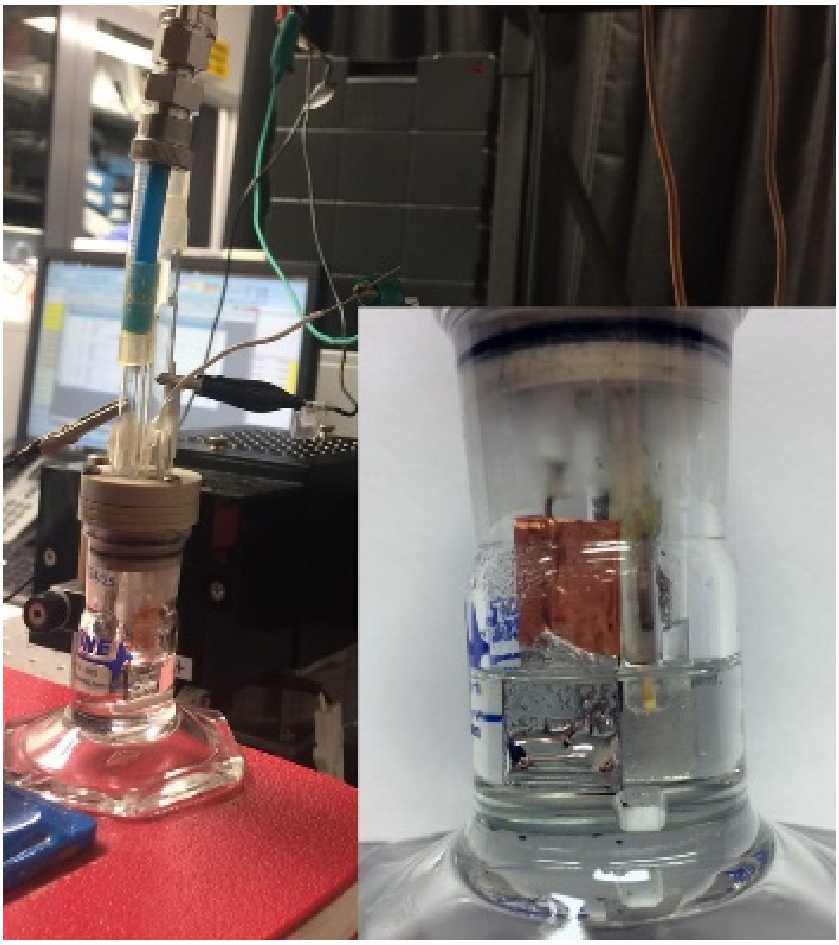

Supplementary Figure 15. Photograph of the electrochemical cell used for gas chromatography analysis.

Supplementary Discussions:

Further consideration regarding *operando* Raman spectroscopy and the deduced reaction mechanism:

Liquid metals provide an oxygen free environment within their center. The reason for this is that any oxygen molecules that would dissolve within the metal would instantaneously react and form a metal oxide. As such the interior of the liquid metal is by default devoid of reactive oxygen species. Furthermore, metal oxides are inherently insoluble in liquid metals. These considerations, together with the self-terminating Cabrera-Mott growth process being dominant for metal oxidation at ambient temperatures, lead to oxidation being limited to the top few nm of the liquid metal air interface. As such Liquid metals are oxygen free and any metallic particle suspended within is protected by the liquid metal matrix. This is what was observed for the Ce rich liquid metal alloys.

Due to the inherent immiscibility of liquid metals with non-metallic materials, the catalytic process involves by default the interface of the catalyst which is dominated by Ce_2O_3. Stripping voltammetry revealed that the reduction process of Ce^{3+} ions occurs on the liquid metal electrode at -1.3 V vs Ag/Ag^+. This potential coincides with our onset potential for electrocatalysis. As such it is reasonable to conclude that the Ce_2O_3 that is usually present on the electrode surface when no potential is applied will be partially reduced to zero valent Ce^0. This Ce^0 is believed to be the active catalytic site. Rather than being stabilized at the interface, the Ce^0 is continuously produced and consumed during the catalytic cycle (see main text). The metallic cerium nanoparticles suspended within the liquid metal clearly facilitate the CO_2 reduction reaction, since an increase of the Ce concentration well beyond the solubility limit is observed to lead to enhanced catalytic activity. We argue that these nanoparticles, which are believed to be abundant throughout the liquid alloy (the concentration is 3% by weight), serve as a cerium metal reservoir that is placed close to the interface.

The peak height of the Ce_2O_3 Raman signal appears to remain relatively constant. Here it is important to recognize the limitations of operando Raman spectroscopy, where absolute signal intensity may vary between individual points due to ripples and bubble formation on the liquid metal surface. A second limitation is that parts of the surface oxide might physically be detached due to bubble formation. At higher applied potential, CO and H_2 are formed as gaseous side products. These bubbles deform the liquid metal surface and may

delaminate oxide flakes during catalysis. Electronically detached oxide flaxes will not participate in the reaction but will contribute to the operando Raman spectra.

When comparing the peak height to other prominent peaks, it appears to vary slightly in magnitude but remains clearly visible for all applied potentials. This is not surprising since the reduction process of the surface oxide is expected to not occur homogeneously across the entire surface, but may be rather patchy. Once a catalytic site has been activated, it will support electocatalysis and the electrocatalytic reduction of CO_2 to carbonaceous materials. Due to the kinetic laws that govern electrochemistry (i.e. the reaction rate increasing exponentially with increasing overpotential), only few active sites are necessary to support a significant current. As such not all of the surface Ce_2O_3 needs to be reduced to Ce^0 to support increasing reaction rates at higher applied potentials, instead, already activated sites simply become even more active with increased overpotential. The detection of a significant CeO_2 peak during operando Raman spectroscopy, however, clearly highlights that a substantial number of active sites have been created.

Supplementary References

1 Wu, Y. *et al.* Electroreduction of CO_2 Catalyzed by a Heterogenized Zn–Porphyrin Complex with a Redox-Innocent Metal Center. *ACS Cent. Sci.* **3**, 847-852, (2017).

2 Mohamed, E. A., Zahran, Z. N. & Naruta, Y. Efficient Heterogeneous CO_2 to CO Conversion with a Phosphonic Acid Fabricated Cofacial Iron Porphyrin Dimer. *Chem. Mater.* **29**, 7140-7150, (2017).

3 Ye, L. *et al.* Highly oriented MOF thin film-based electrocatalytic device for the reduction of CO_2 to CO exhibiting high faradaic efficiency. *J. Mater. Chem. A* **4**, 15320-15326, (2016).

4 Aljabour, A. *et al.* Nanofibrous cobalt oxide for electrocatalysis of CO_2 reduction to carbon monoxide and formate in an acetonitrile-water electrolyte solution. *Appl Catal B.* **229**, 163-170, (2018).

5 Zhang, Z. *et al.* Rational Design of Bi Nanoparticles for Efficient Electrochemical CO_2 Reduction: The Elucidation of Size and Surface Condition Effects. *ACS Catal.* **6**, 6255-6264, (2016).

6 Zhang, S. *et al.* Polymer-supported CuPd nanoalloy as a synergistic catalyst for electrocatalytic reduction of carbon dioxide to methane. *Proc. Natl. Acad. Sci. U.S.A.* **112**, 15809-15814, (2015).

7 Zhu, Q. *et al.* Efficient Reduction of CO2 into Formic Acid on a Lead or Tin Electrode using an Ionic Liquid Catholyte Mixture. *Angew. Chem.* **128**, 9158-9162, (2016).

8 Huan, T. N. *et al.* Cu/Cu_2O Electrodes and CO_2 Reduction to Formic Acid: Effects of Organic Additives on Surface Morphology and Activity. *Chem. Eur. J.* **22**, 14029-14035, (2016).

9 Lu, L. *et al.* Selective electroreduction of carbon dioxide to formic acid on electrodeposited SnO_2@N-doped porous carbon catalysts. *Sci. China Chem.* **61**, 228-235, (2018).

10 Huan, T. N. *et al.* From molecular copper complexes to composite electrocatalytic materials for selective reduction of CO_2 to formic acid. *J. Mater. Chem. A* **3**, 3901-3907, (2015).
11 Costentin, C., Drouet, S., Robert, M. & Savéant, J.-M. Turnover numbers, turnover frequencies, and overpotential in molecular catalysis of electrochemical reactions. Cyclic voltammetry and preparative-scale electrolysis. *J. Am. Chem. Soc.* **134**, 11235-11242, (2012).
12 Zhang, S. *et al.* Polymer-supported CuPd nanoalloy as a synergistic catalyst for electrocatalytic reduction of carbon dioxide to methane. *Proc. Natl. Acad. Sci. U.S.A.***112**, 15809-15814, (2015).
13 Huan, T. N. *et al.* From molecular copper complexes to composite electrocatalytic materials for selective reduction of CO_2 to formic acid. *J. Mater. Chem. A* **3**, 3901-3907, (2015).
